# Myocardial Bridging

**DOI:** 10.5935/1678-9741.20150082

**Published:** 2016

**Authors:** Shi-Min Yuan

**Affiliations:** 1 The First Hospital of Putian, Teaching Hospital, Fujian Medical University, Fujian Province, China.

**Keywords:** Atherosclerosis, Cardiac Surgical Procedures, Myocardial Bridging, Myocardial Infarction

## Abstract

Myocardial bridging is rare. Myocardial bridges are most commonly localized in the middle segment of the left anterior descending coronary artery. The anatomic features of the bridges vary significantly. Alterations of the endothelial morphology and the vasoactive agents impact on the progression of atherosclerosis of myocardial bridging. Patients may present with chest pain, myocardial infarction, arrhythmia and even sudden death. Patients who respond poorly to the medical treatment with β-blockers warrant a surgical intervention. Myotomy is a preferred surgical procedure for the symptomatic patients. Coronary stent deployment has been in limited use due to the unsatisfactory long-term results.

## INTRODUCTION

Myocardial bridging is systolic compression of a tunneled coronary artery by the overlying myocardial tissue, which disappears completely during diastole^[1]^. This coronary anomaly is usually a benign condition, but it can be associated with a series of serious cardiac events, such as myocardial infarction, arrhythmia and sudden death^[2]^. Male predominance has been noted in large series of myocardial bridging patients^[3]^. The prevalence varies between 0.5% and 86% among different studies with a much higher rate at autopsy than in angiography^[4]^.

Myocardial bridges are most commonly localized in the middle segment of the left anterior descending coronary artery. Diagonal and marginal branches can be involved in 18% and 40% of cases, respectively. Myocardial bridging can be single or multiple. The multiple ones can occur in a same or different coronary artery or their branches^[5]^. Ferreira et al.^[3]^ divided the myocardial bridging into two types: superficial and deep muscle types. The former does not constrict the coronary flow during systole; whereas the latter may compress the coronary artery, reduce the flow and induce myocardial ischemia. Noble et al.^[6]^ categorized the systolic coronary narrowing into three classes: Class 1 (systolic coronary narrowing <50%), Class 2 (systolic coronary narrowing 50-75%) and Class 3 (systolic coronary narrowing >75%).

The anatomic features of the bridges vary significantly with a length of 2.3-42.8 mm, a thickness of 1.0-3.8 mm and an angle between long axis of muscle fibers and long axis of the crossed vessel of 5°-90°^[7]^. It was reported that the mean length of the bridges was 14.64±9.03 mm and the mean thickness was 1.23±1.32 mm^[8]^.

## PATHOPHYSIOLOGY

The pathophysiology of myocardial bridging is insufficiently understood. Myocardial bridges are usually small and have no clinical significance. The segment proximal to the region of the myocardial bridging has been associated with atherosclerosis rather than the myocardial bridging segment itself^[9]^. Both hemodynamic and structural changes, such as blood flow disturbance, myocardial malperfusion, deposits of lipids and mucopolysaccharides and elastic damages, can be noted in the coronary artery segment proximal to a myocardial bridge. All these changes predispose to formation of atherosclerotic plaques in the intima of the coronary artery segment. Obviously, myocardial bridging is associated with degenerations of both myocardium and coronary artery^[10]^. The pathophysiological studies indicated significant impairment of coronary blood flow based on bridge obstruction as the underlying mechanism of sudden cardiac death in the patients with myocardial bridging^[11]^.

Alterations of the endothelial morphology along with disturbance of blood flow and high wall stress proximal to myocardial bridging have been identified as the main causes of atherosclerosis in the segment proximal to the bridge^[12]^. In addition, vasoactive agents (endothelin-1, endothelial nitric oxide synthase and angiotensin-converting enzyme) have been shown to be present in higher concentrations in the proximal portion of the myocardial bridging artery in comparison to the myocardial bridging segment, thereby supporting the mechanisms of atherosclerosis found in the proximal segment^[13]^.

The mechanical stress caused by systolic narrowing at the myocardial bridging segment may result in endothelial damage, which, conversely, may induce platelet aggregation, coronary vasospasm and eventually acute coronary syndrome^[14]^. Endothelial damage, vasospasm and atherosclerotic processes developing in the proximal portion of the bridging segment are alternative causes of ischemia. Hostiuc et al.^[15]^ have reported significant myocardial fibrosis and interstitial edema in the myocardial bridging segment in patients with later sudden death.

## CLINICAL MANIFESTATION

The clinical manifestations of the patients with myocardial bridging can appear in two ways: 1) by contraction of myocardial bridge fibers and direct compression of the tunneled segment; or 2) by stimulation and acceleration of atherosclerosis in the segment proximal to the myocardial bridging^[8]^. Significant myocardial bridging is often associated with total or subtotal occlusion of the left anterior descending coronary artery during systole. In patients with myocardial bridging, symptoms often manifest during exercise and with tachycardia^[2]^. The patient may manifest chest squeezing at rest^[16]^. In young patients with myocardial bridging, they may have an acute anterior myocardial infarction due to a subtotal occlusion of the mid-left anterior descending coronary artery caused by myocardial bridging^[17]^. Symptomatic patients with myocardial bridging may present with myocardial ischemia, acute coronary syndromes, coronary spasm, exercise-induced dysrhythmias (such as supraventricular tachycardia, ventricular tachycardia, or atrioventricular block), myocardial stunning, transient ventricular dysfunction, syncope, or even sudden death^[18]^. When myocardial bridging is associated with heart valve disorder or cardiomyopathies, the patients' symptoms can be different. Sustained elevated troponin levels suggested the presence of myocardial ischemia^[19]^.

Coronary angiography, intracoronary Doppler imaging and intravascular ultrasonography have documented a characteristic diastolic flow disturbance. Three-dimensional speckle-tracking echocardiography can detect subtle myocardial dysfunction in patients with myocardial bridging in terms of amplitudes of longitudinal, circumferential and radial strains^[20]^. Multiple-slice computed tomography, stress single-photon emission computed tomography and stress echocardiography are helpful for the diagnosis of myocardial bridging. Multislice spiral computed tomography defines bridges as segments surrounded by myocardium and is more helpful in identifying hemodynamically significant myocardial bridging. On angiography, diagnosis depends on the change in diameter between systole and diastole within the bridged coronary segment. A significant "milking effect" is present when there is ≥70% reduction in minimal luminal diameter during systole and persistent ≥35% reduction in minimal luminal diameter during mid-to-late diastole. Systolic narrowing at the bridge can be accentuated by intracoronary injection of nitroglycerin by vasodilating adjacent non-bridged coronary segments^[18]^.

Metrological studies have revealed angiographically a systolic diameter reduction of 80.6±9.2% and a persistent diastolic reduction of 35.3±11% within the tunneled segment. Diastolic flow velocities within the bridging segment were much higher than those in the proximal and distal portions of the bridging segment. Coronary flow reserve distal to the bridge was 2.5±0.5^[21]^. The tunneled artery is significantly thinner (66.3 µm) than that of the proximal segment^[22]^. The intracoronary Doppler revealed a lumen reduction during systole secondary to systolic compression of the myocardial bridge was 36.4±8.8%^[12]^.

## MANAGEMENT AND PROGNOSIS

### Pharmacological therapy

Because the patients with myocardial bridging are at increased risk for atherosclerosis, antiplatelet therapy should be considered. For symptomatic patients, β-blockers remain the main conservative treatment and they may relieve the patients from hemodynamic impairment caused by the myocardial bridging by decreasing the heart rate, increasing the diastolic coronary filling and decreasing the contractility and compression of the coronary arteries. Calcium channel blockers may have vasodilatory effects beneficial for the concomitant vasospasm. It is advised that vasodilating agents including nitroglycerin should be cautiously prescribed for the patients with myocardial bridging. Nitrates may exacerbate symptoms by intensifying systolic compression of the bridged segment and vasodilating segments proximal to the bridge, and therefore vasodilators should be avoided unless there is significant coexisting coronary vasospasm.

### Percutaneous coronary intervention

Stent implantation in symptomatic patients with myocardial bridges may alleviate systolic coronary compression and improves patients' conditions; however, potential complications of coronary artery and stent itself in relation to stent deployment have limited its use. However, drug-eluting stents may be preferable for the avoidance of future reintervention^[18]^.

### Surgery

Surgical intervention involves either supra-arterial myotomy or coronary artery bypass. Coronary artery bypass is indicated for the patients with extensive (>25 mm) or deep (>5 mm) myocardial bridging or when the tunneled coronary segment is unlikely to be decompressed completely in diastole^[18]^. The potential complications of myotomy include wall perforation, ventricular aneurysm formation and postoperative bleeding^[18]^; while the major concerns of coronary artery bypass in the patients with myocardial bridge is lower freedom of angina and graft failure^[23]^. Rezayat et al.^[24]^ performed surgical myotomy for myocardial bridging in 26 patients and in one of the patients postoperative residual narrowing of the left anterior descending coronary artery was noted as the only complication of the patient cohort. Zhu et al.^[25]^ reported their retrospective results of mini-incision myotomy for myocardial bridging of the left anterior descending coronary artery with a systolic narrowing extent of the bridging >60% in all 11 patients. Via a lower partial mid-sternotomy, 10 patients received surgical myotomy with one of them complicated with coronary artery impairment and off-pump coronary artery bypass was performed in this patient. Another patient had myotomy and concurrent repair of left anterior descending coronary artery-pulmonary artery fistula without pump. During the 2-51 month follow-up, one patient with myotomy having recurrent angina received medical treatment and the patient with coronary artery bypass also had recurrent angina and a coronary stent was deployed. Moreover, myotomy through heart-port access for myocardial bridging has also been reported^[20]^.

## CONCLUSION

Myocardial bridging is most often located in the left anterior descending coronary artery. It can be associated with a series of severe cardiovascular events, such as myocardial infarction, arrhythmia and sudden death. Symptomatic patients should be treated conservatively, interventionally or surgically depending on the patients' conditions. Myotomy is a preferred surgical procedure for relieving the patients' symptoms, improving the coronary flow and alleviating the coronary artery compression secondary to myocardial bridging.

**Table:** 

**Author's roles & responsibilities**	
SMY	Study conception and design; analysis and/or interpretation of data; manuscript writing, final approval of the manuscript
